# Subvoxel Accurate Graph Search Using Non-Euclidean Graph Space

**DOI:** 10.1371/journal.pone.0107763

**Published:** 2014-10-14

**Authors:** Michael D. Abràmoff, Xiaodong Wu, Kyungmoo Lee, Li Tang

**Affiliations:** 1 Department of Ophthalmology and Visual Sciences, Stephen A Wynn Institute for Vision Research, Department of Biomedical Engineering, and Department of Electrical and Computer Engineering, University of Iowa, Iowa City, Iowa, United States of America; 2 Iowa City Veterans Administration Medical Center, Iowa City, Iowa, United States of America; 3 Department of Electrical and Computer Engineering, Department of Radiation Oncology, University of Iowa, Iowa City, Iowa, United States of America; 4 Department of Electrical and Computer Engineering, University of Iowa, Iowa City, Iowa, United States of America; 5 Department of Ophthalmology and Visual Sciences, University of Iowa, Iowa City, Iowa, United States of America; Institute of Automation, Chinese Academy of Sciences, China

## Abstract

Graph search is attractive for the quantitative analysis of volumetric medical images, and especially for layered tissues, because it allows globally optimal solutions in low-order polynomial time. However, because nodes of graphs typically encode evenly distributed voxels of the volume with arcs connecting orthogonally sampled voxels in Euclidean space, segmentation cannot achieve greater precision than a single unit, i.e. the distance between two adjoining nodes, and partial volume effects are ignored. We generalize the graph to non-Euclidean space by allowing non-equidistant spacing between nodes, so that subvoxel accurate segmentation is achievable. Because the number of nodes and edges in the graph remains the same, running time and memory use are similar, while all the advantages of graph search, including global optimality and computational efficiency, are retained. A deformation field calculated from the volume data adaptively changes regional node density so that node density varies with the inverse of the expected cost. We validated our approach using optical coherence tomography (OCT) images of the retina and 3-D MR of the arterial wall, and achieved statistically significant increased accuracy. Our approach allows improved accuracy in volume data acquired with the same hardware, and also, preserved accuracy with lower resolution, more cost-effective, image acquisition equipment. The method is not limited to any specific imaging modality and readily extensible to higher dimensions.

## Introduction

Object segmentation has been widely used in image understanding and object recognition for decades [1], especially in quantitative analysis of volumetric medical images [2], [3]. Typically, tissues are organized in layers, and to segment their boundaries or surfaces, the segmentation problem can be transformed into the problem of computing a minimum closed set in a node-weighted directed graph [4], [5]. Every node represents a single voxel, while the graph represents the voxel grid. The optimal surface corresponds to the upper envelope of the minimum closed set of nodes. Because volume data is typically represented as an orthogonal matrix of intensities, the surface segmentation can not achieve greater precision than a single unit, or the distance between two adjoining nodes in the graph.

However, higher segmentation accuracy than unit allows better diagnosis and treatment of disease, and equal segmentation accuracy with lower resolution image acquisition hardware allows more cost-effective imaging.

Graph techniques, a generalization of 2D shortest path-based segmentation [6], [7], provide globally optimal solutions with respect to a cost function for surface segmentation in three-dimensional volumes in polynomial time [5]. They allow incorporation of various feasibility constraints and regional information [8] for simultaneous segmentation of multiple surfaces [9]. Additional terms in the cost function make it possible to penalize local shape or surface distance changes by learning the expected model during a training process [10]. Unfolding techniques were developed to segment objects with complex shapes, such as knee bone and cartilage [11], heart [12], pulmonary airway [2], vascular trees [13], retinal lesions [14] and retinal vessels [15]. However, all techniques use a graph in Euclidean space with nodes corresponding to the center of evenly distributed voxels, thus limiting segmentation precision to a single unit.

Volumetric images are formed by discretizing into voxels the continuous intensity function sampled by sensors, resulting in partial volume effects [16], [17]. Partial volume effects contain additional information that can potentially be exploited by graph techniques. However, they are ignored if the intensity, or a derivative thereof such as a gradient, measured at the center of each voxel, is assigned to nodes as their costs in a graph in Euclidean space. By generalizing the graph to non-Euclidean space, i.e. allow non-equidistant non-orthogonal spacing between nodes on any dimension, this previously ignored information can be used, while all the advantages of graph techniques sketched above are retained, including global optimality and computational efficiency. We define such a graph search as non-Euclidean graph search.

**Figure 1:**
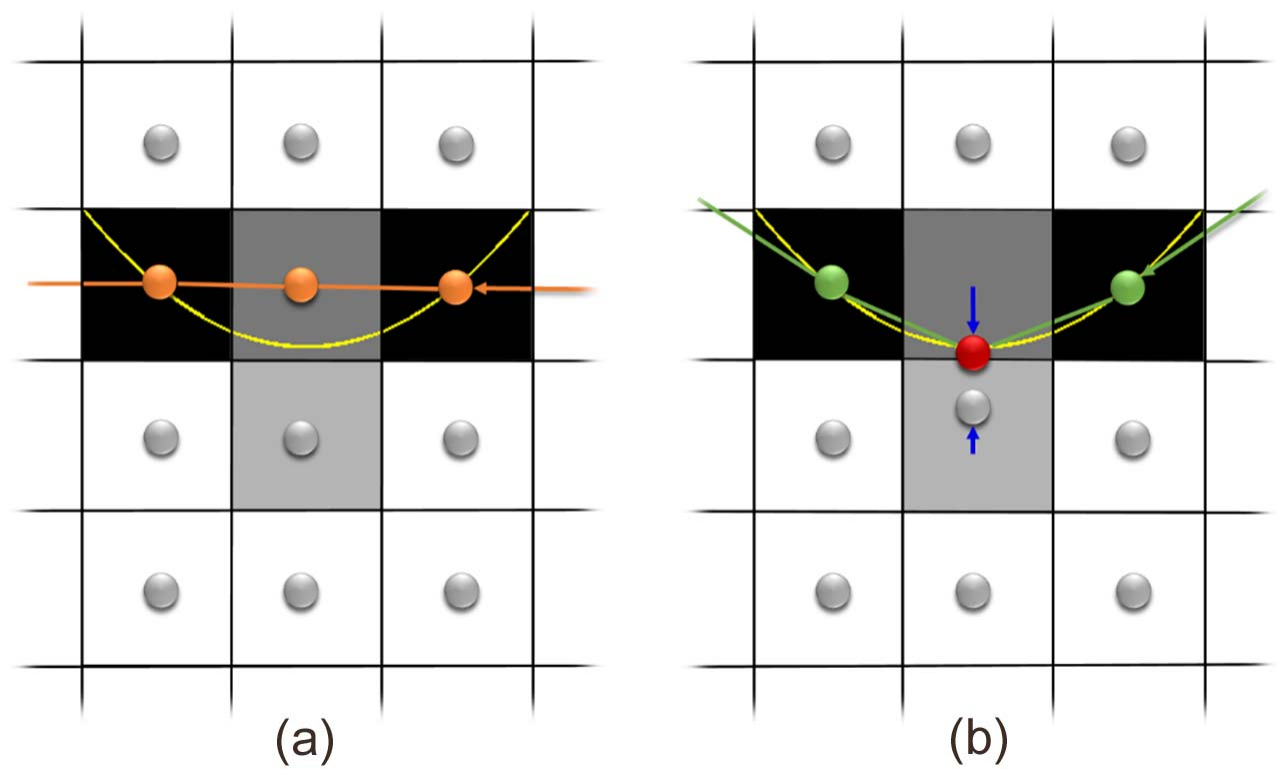
Graphs in Non-Euclidean space allow surface localization with subvoxel accuracy. (a) the graph in conventional Euclidean space allows graph search to find the lowest cost path (darkest voxel intensity) with voxel accuracy, compared to the yellow true surface; (b) the Non-Euclidean graph allows the green surface to be segmented through the lowest cost path with subvoxel accuracy, after the deformation field derived from the volume data (in this 2D example, derived from the 8-neighborhood) is applied to the two central nodes (blue arrows). Note the partial volume effect compared to their neighboring voxels, indicating that the actual center of low cost path is below the center of the upper dark voxel, as also indicated by the yellow true surface.

Specifically, we apply a non-Euclidean deformation in constructing the graph using a displacement field obtained directly from the volume data. This principle is sketched in Fig. 1 using a simplified 2D example. The density of nodes thus increases at regions where salient transitions of image properties are more likely to occur, while the displacement of each node is confined to the same voxel. Overall the complexity of the graph structure in terms of the number of nodes and arcs is unchanged, so that memory requirements and running time are unchanged except for the computation of the deformation field. The graph space is deformed in a Euclidean way. However, since we do not define a distance metric between nodes in the graph, we refer to this graph structure as a non-Euclidean graph. Effectively, the deformation field adaptively changes the regional node density so that this is higher in regions where the target boundary is expected to pass through, and lower in the remaining region, while retaining the overall node density.

The purpose of this study is to introduce and describe this novel approach, and validate its increased accuracy by comparing it to conventional graph search segmentation of down-sampled optical coherence tomography (OCT) volumes of the retina and magnetic resonance imaging (MRI) of the carotid vessel wall. The non-Euclidean graph approach turns out to have superior segmentation accuracy over conventional graph search. We use downsampled volumes as the input data for these comparative studies, while the accurately segmented high resolution surfaces from the original, higher resolution volumes serve as the reference standard. The approach is general and can be adapted to other imaging modalities as well.

## Methods

### 0.1 Euclidean Space Graph Representation of Layered Tissues

Let 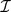 be a given 3D volumetric image with a size of 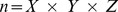. For each 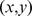 pair, 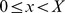 and 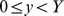, the voxels with different 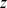-coordinates, that is, the voxel subset 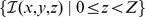, forms a voxel-column parallel to the 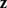-axis, denoted by 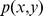. Two voxel-columns are neighboring if their 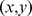-coordinates satisfy some neighborhood condition. For example, under the 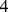-neighboring setting, the voxel-column 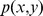 is neighboring to the voxel-column 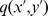 if 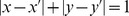. Henceforth, a model of the 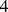-neighboring setting is used; this simple model can be easily extended to other neighborhood conditions. Each of the target terrain-like surfaces contains one and only one voxel in each column of 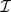 (Fig. 1 (a)).

The feasibility of the target surfaces is governed by the surface smoothness and separation constraints. The surface smoothness constraint is specified by two smoothness parameters, 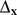 and 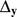, which define the maximum allowed change in the 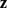-coordinate of a surface along each unit distance change in the 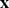 and 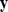 dimensions, respectively. If 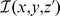 and 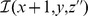 (resp., 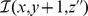) are two (neighboring) voxels on a feasible surface, then 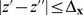 (resp., 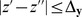).

In multiple surface detection, the surface separation constraint specifies the minimum and maximum distances along the **z**-dimension between any pair of the target surfaces of interest. Each voxel 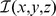 has a real-valued cost 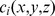 for each sought surface 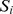, which is inverse to the likelihood that the voxel is on the surface. For a given integer 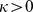, the surface segmentation problem seeks to identify an optimal set of 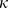 surfaces with minimum total cost by summing the costs associated with all voxels on all sought surfaces.

### 0.2 Conventional Euclidean Space Graph Search Segmentation

Based on previously reported graph-theoretic segmentation techniques [2], [4], [5], [10], the surface segmentation problem in 3D volumetric images 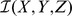 is formulated as computing a minimum closed set in a node-weighted directed graph. For single surface segmentation (i.e. 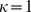), the graph 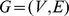 consists of a set of nodes 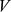 and a set of arcs 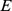 connecting pairs of nodes. Every node 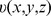 is created from exactly one voxel of 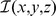. The subset of nodes corresponding to a voxel-column 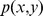 of voxels in 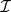 forms a node-column, denoted by 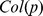. Arcs connecting two neighboring nodes on the same column are intra-column arcs while those connecting two nodes from neighboring columns are inter-column arcs. The intra-column arcs pointing downward enforce the constraint that a feasible surface 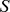 intersects each voxel-column only once. The inter-column arcs impose smoothness constraints between neighboring voxel-columns.

A closed set is a subset of nodes which have no arcs leaving the set. Each non-empty closed set uniquely defines a feasible surface 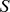 in 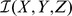. In order to find a minimum closed set 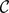 whose total cost is the summation of costs of all nodes contained in the region bounded by surface 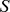, the weight 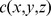 of each node 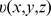 is assigned its original cost 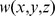 minus the cost of the node immediately below it, i.e. cost 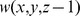 of node 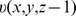 [2], [4], [5]:
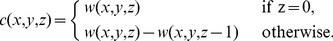
(1)


By solving the minimum closed set problem, each node gets a binary label 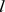 indicating if it is contained in 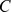. The upper envelope of 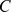 corresponds exactly to the optimal surface with the same minimum total cost in the original graph.

For simultaneously segmenting 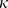 interrelated surfaces, a similar graph structure is duplicated 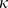 times with respect to a particular surface. The costs assigned to each sub-graph reflects whether the data favor the nodes belonging to that certain surface. Inter-subgraph arcs are created to connect two nodes from corresponding columns of different sub-graphs, imposing neighboring surface separation constraints. The total cost is the summation of the cost in 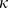 sub-graphs.

### 0.3 Steps in Non-Euclidean Graph Search

**Figure 2:**
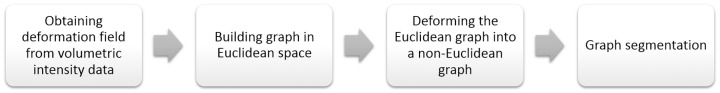
Non-Euclidean graph search proceeds in four steps.

Non-Euclidean graph search proceeds in the following steps (Fig. 2):

1. Obtain a deformation field from the volumetric intensity data;
2. Build the graph in Euclidean space as in Section 0.2;
3. Deform the Euclidean space graph into a non-Euclidean graph using the deformation field;
4. Graph segmentation as in Section 0.1.

It is worth noting that after deformation, the globally optimal solution is searched for in the new non-Euclidean graph space, which fundamentally distinguishes our approach from those local approaches, such as the one proposed in [18]. It starts from a regular segmentation and interprets partial belongingness of graph elements using cuts with sub-edge precision. The improvements are essentially local adjustments in contrast to our globally optimal solution.

#### 0.3.1 Compute Deformation Field from Volumetric Data

A shift of evenly distributed voxels to a deformed graph space, defined as a non-Euclidean deformation field 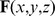 acting on the center of each voxel 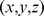:
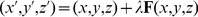
(2)where 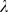 represents a normalization factor. For the worst case, the error introduced by a voxel is equal to half of the voxel size 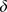. Therefore 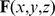 is normalized such that the maximum deformation is equal to 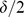:



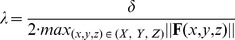
(3)Please note that the deformation in this study is not refer to the deformation from a source to a target. It is a deformation along a predefined direction up to a chosen allowable maximum.

The deformation field 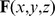 can be calculated from the negative diffusion of the gradient vectors of cost 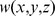 derived from intensity volume data 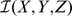 [19]:
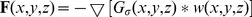
(4)where 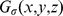 is a regularization imposed by a 3D Gaussian kernel with standard deviation of 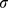, the symbols 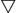 and 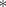 represent the gradient and convolution operators respectively. Other methods can be used to obtain 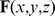 as well, such as the gradient vector flow field (GVF) [19], though we consider it less important as long as a deformation field 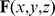 is obtained. As a feature-preserving diffusion of the gradient, GVF is defined as the vector field that minimizing the following energy function:



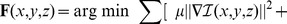


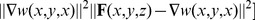
(5)


The parameter 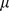 regularizes the importance of the two terms contributing to the energy function [19].

**Figure 3:**
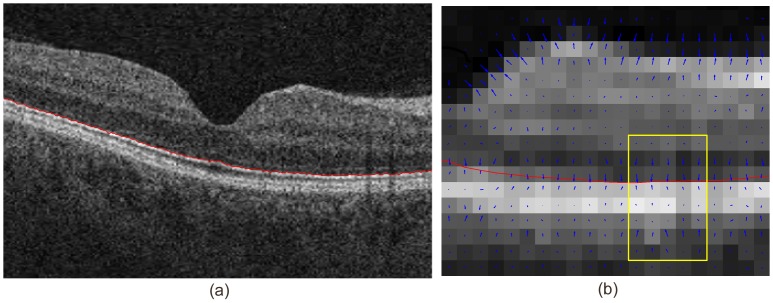
2D deformation field of one B-scan in a OCT volume. (a) A single boundary was delineated in one B-scan of a OCT volume; (b) The 2D deformation field of the re-sampled B-scan indicates the actual boundary location within each voxel under partial volume effects.

Fig. 3 shows the deformation field in a single B-scan of an OCT volume, calculated according to Eq. 4. In Fig. 3 (a), a single boundary 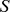 of high resolution image 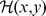 (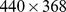 pixels) was delineated by a red line. The B-scan 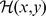 was down-sampled to 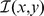 by ten times to 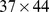 pixels to mimic the imaging and discretization process of the continuous tissue intensity function. The down-sampling was performed by first reconstructing a continuous signal from the original discrete signal by bicubic interpolation [20]. Then a low-pass anti-aliasing filter was applied to the reconstructed continuous signal. Finally the reconstructed, antialiased continuous signal was re-sampled at the desired new sampling rate to get the down-sampled signal, i.e. down-sampled B-scan in this case. The boundary surface 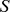 was mapped from the high resolution to its location in the re-sampled B-scan 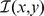 in lower resolution. Part of a zoomed-in view of 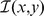 was shown in Fig. 3 (b) with its 2D deformation vectors 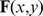 displayed as blue arrows starting from the center of each pixel 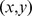.

**Figure 4:**
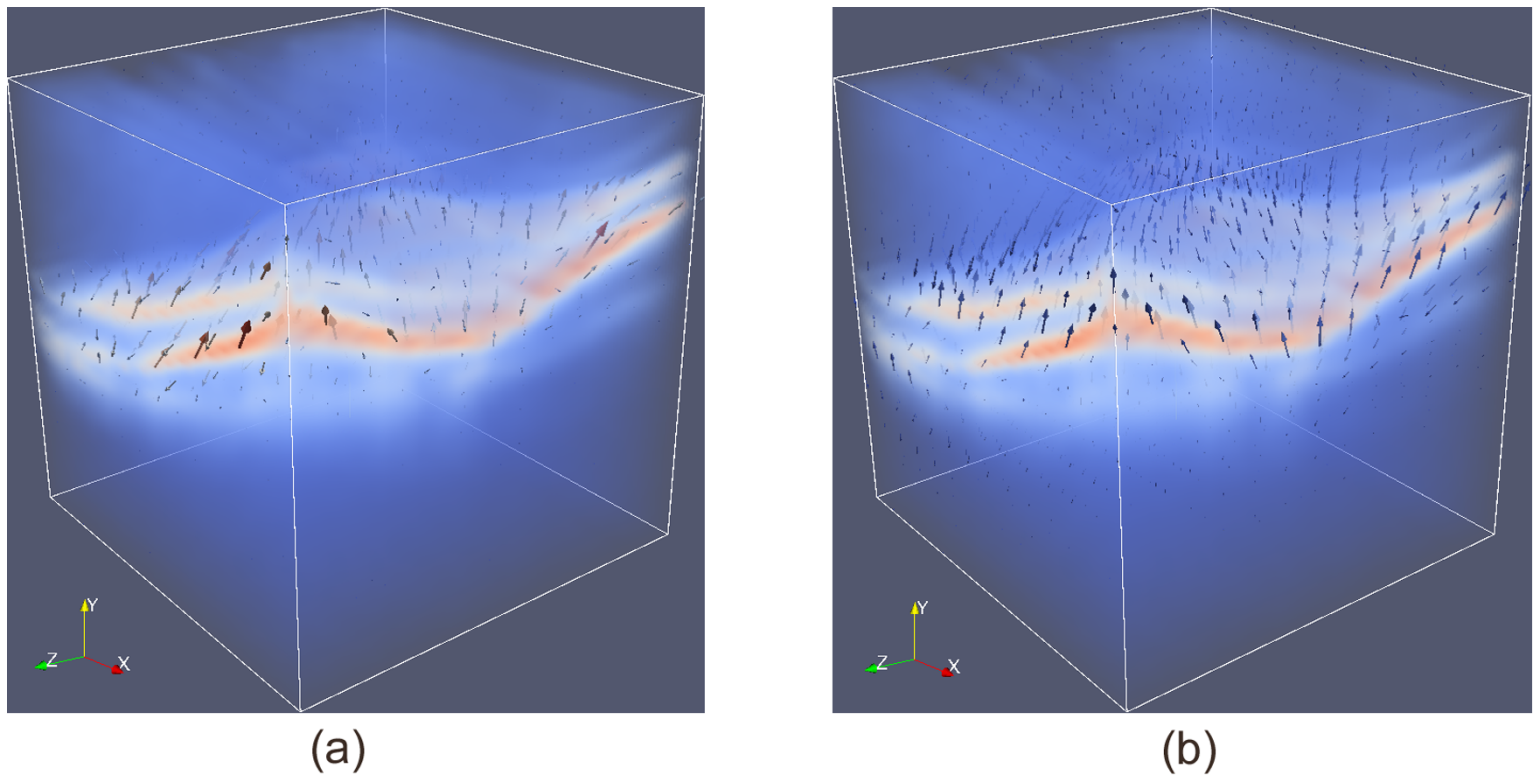
Impact of regularization on the deformation field of a 3D OCT volume. (a) Deformation field before regularization; (b) Deformation field after regularization.

Fig. 4 demonstrates the effect of regularization 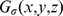 in Eq. 4 on the deformation field of a 3D OCT volume, where the spacing along each dimension was made equal to give an isotropic illustration. Red voxels indicate high intensity values while blue voxels indicate low intensity values in the original volume. The impact of parameter 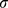 in Eq. 4 includes the capture range towards the desired surface and its localization accuracy.

#### 0.3.2 Content Based Graph Construction

**Figure 5:**
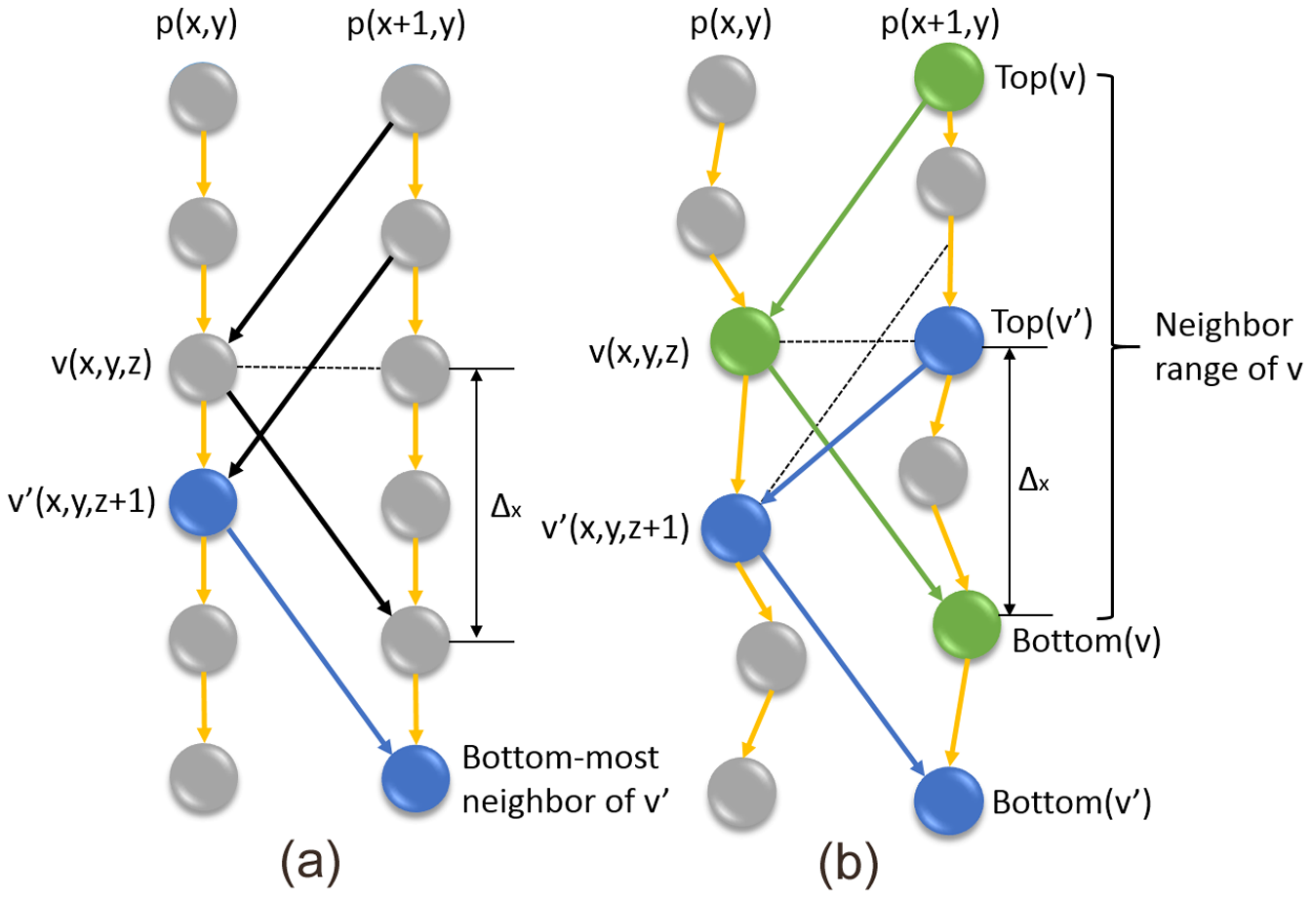
Structure of conventional graph (a) and non-Euclidean deformed graph (b). In (a), node 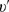 and its bottom-most neighbor are shown in dark blue. In (b), node 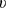, 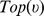, 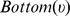 are shown in green and node 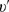, 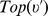, 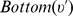 are shown in dark blue.

We first construct a directed graph 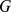 from the input image 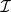 using the conventional graph search methods [2], [4], [5], as briefly described in Section 0.1 (refer to Fig. 5 (a)). Then the deformation field 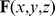 is applied to deform each node in 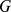 with Eq. 2. That is, each node 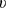 at 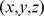 is deformed to 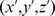 with 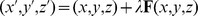. The deformation operation may violate the surface smoothness constraints. Note that the deformed graph nodes corresponding to the voxels on a voxel-column 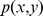 still form a node-column 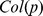 in 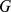, and each target surface 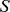 is monotonic with respect to each (deformed) node-column in 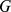. The intra-column arcs thus need not to be modified. In the following, we will focus on the adjustment to the inter-column (inter-subgraph) arcs to incorporate the surface smoothness (separation) constraints.

For the surface smoothness constraints, consider two adjacent columns 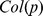 and 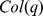 in 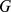. For any two nodes 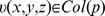 and 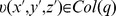, if 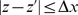 (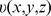 and 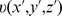 are adjacent along the 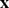-dimension) or 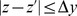 (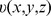 and 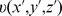 are adjacent along the 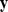-dimension), then the corresponding voxel points 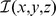 and 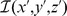 can be both on the target surface. By abuse of notation, we say that a node 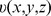 is on the surface, which means that its corresponding voxel is on the surface.

To explore the self-closure structure [4], [5] of the surface segmentation problem, we define the bottom-most neighbor and the top-most neighbor of a node 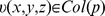 on its adjacent column 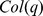: the bottom-most (resp., top-most) neighbor of 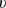, denoted by 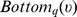 (resp., 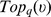), is a node on 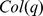 with the smallest (resp., largest) 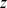-coordinate that can be on the target surface together with 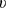. In the case that the adjacent column is clear, we can simply denote the bottom-most (resp., top-most) neighbor by 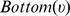 (resp., 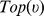) (Fig. 5). Thus, each node 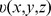 can interact with a range of nodes between 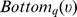 and 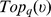 on 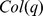, called the neighbor range of 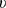 on 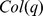.

In the conventional graph search method, the number of nodes in the neighbor range for every node of 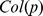 is the same [2], which, however, may differ in the deformed graph space. Fortunately, all the neighbor ranges of nodes on any column 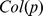 are properly ordered [4], [5]: for any nodes 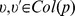, if 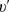 is “above” 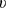 (that is, the 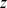-coordinate of 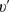 is larger than that of 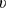), then 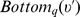 and 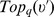 are no “lower” than 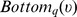 and 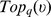 (with respect to the 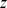-coordinates), respectively, on the adjacent column 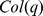.

Denote by 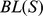 all the nodes on or below a surface 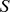. The proper ordering of the neighbor ranges admits the intra-layer self-closure structure: For any feasible surface 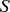, the bottom-most neighbors of every node in 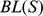 are contained within 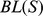.

This is the very property that connects the optimal surface segmentation to the minimum-cost closed set problem. In content based graph construction, two types of arcs, intra-column arcs and inter-column arcs, are illustrated in Fig. 5. The intra-column arcs (yellow arrows) make sure that all nodes below a given node are also included in the closed set, which enforce the monotonicity of the target surface. The inter-column arcs impose the smoothness constraints by directing to the bottom-most neighbors of a given node. If simply the nodes estimated in the initially constructed graph are deformed (i.e., the edges connecting the node pairs are retained), the resulting graph may not correctly impose the surface smoothness constraints or the surface separation constraints, due to the non-uniform shifts of the nodes.

The same strategy can be used to extend the method to simultaneously detect 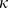 interrelated surfaces with surface separation constraints. The surface separation constraints in a *d*-D image can be viewed as the surface smoothness constraints of a 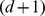-D image, consisting of a stack of 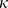 *d*-D images. Segmenting an optimal set of 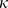 surfaces in a *d*-D image is equivalent to the detection of a single optimal surface in a 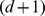-D image.

**Figure 6:**
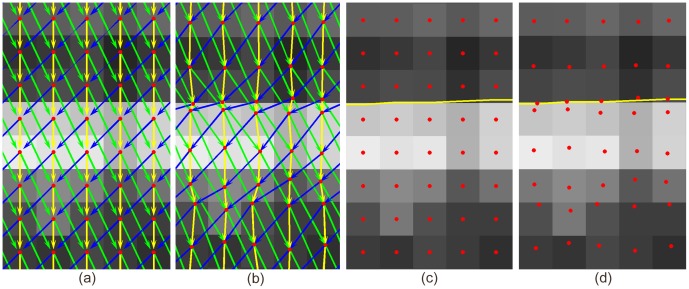
Comparison of conventional graph construction and Non-Euclidean deformed graph model. (a) Conventional graph structure; (b) Non-Euclidean deformed graph model; (c) Node distribution in conventional graph structure; (d) Node distribution in non-Euclidean deformed graph model. Node created within each pixel is shown as red dot. Intra-column arcs are shown as yellow arrows while inter-column arcs are shown as green and blue arrows, which represent smoothness constraints that can be incorporated in a typical graph search framework. The yellow curves indicate the desired dark-to-bright surface location mapped from high resolution B-scan.

Fig. 6 compares the graph in Euclidean space (Fig. 6 (a)) to the non-Euclidean graph after deformation (Fig. 6 (b)) within the region highlighted by the yellow rectangle in Fig. 3 (b). After deformation, a node is created within each voxel at the location indicated by a red dot. Referring to the true boundary shown in Fig. 3 (b), distances between neighboring nodes created in those critical regions are smaller than the distance between voxel centers, allowing subvoxel surface positioning accuracy to be achieved. Fig. 6 (c) and Fig. 6 (d) shows the nodes in the conventional and non-Euclidean deformed graph model, showing that node density is increased near the expected surface, and decreased elsewhere.

The costs associated with each node reflect the properties of the node with respect to the underlying surfaces. For instance, to identify layered tissues - terrain-like surfaces - separated by either dark-to-bright or bright-to-dark transitions, the gradient component along the 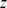-dimension with an opposite orientation can be assigned as the on-surface cost associated with each node for simplicity, derived from convolution of volume 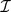 with the first derivative of 3D Gaussian kernel 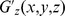 [21]:
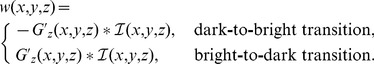
(6)


Other costs can be employed as in conventional graph search [2], [5], which makes it possible to include not only boundary (on-surface cost) but also region (in-region cost) properties.

Since the deformation field 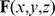 may deform each node away from the voxel center 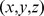, the original cost 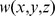 derived from the evenly distributed voxel grid has to be deformed or warped in a similar way, so that the nodes in the non-Euclidean graph space are assigned the correct costs 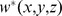 according to their updated locations:
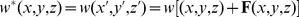
(7)


**Figure 7:**
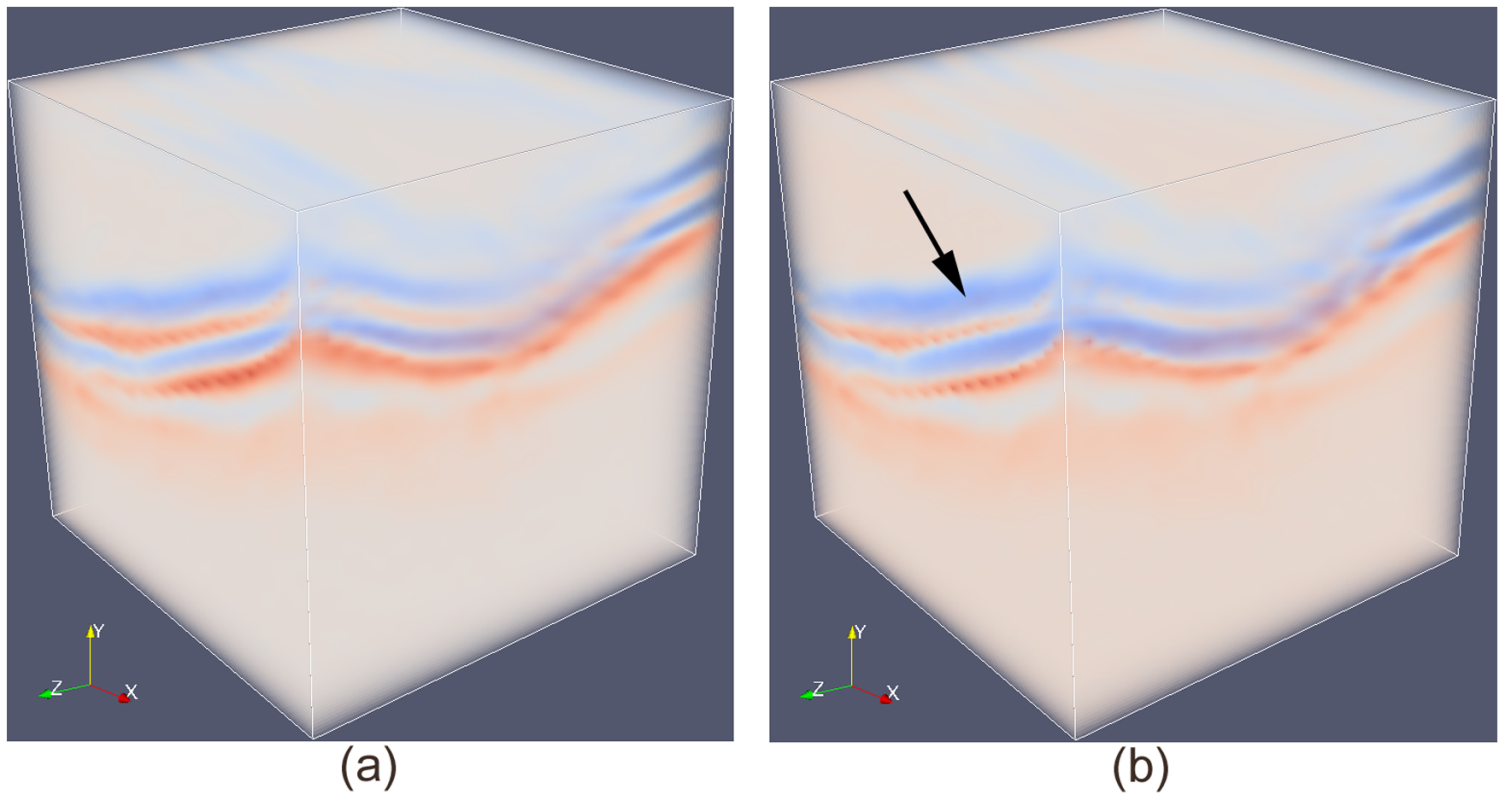
Node density is higher around target surface after non-Euclidean deformation by stretching area of interest as the one indicated by an arrow. (a) Original cost volume, where red voxels indicate high costs while blue voxels indicate low costs; (b) Cost volume after non-Euclidean deformation.

If we plot the new costs as a volume confined on an evenly distributed grid, as is shown in Fig. 7, the low cost regions in blue (indicated by an arrow), i.e. regions with high likelihood of being surface boundaries, are stretched out, which is equivalent to adaptively increasing the local node density in those highly relevant regions as explained in Section 0.3.

## Results

### Intraretinal Surface Segmentation of SD OCT Images

Quantitative analysis of retinal layers is crucial for diagnosis and management of eye and systemic diseases, including diabetic retinopathy, age-related macular degeneration, and glaucoma, as well as hereditary diseases such as Best's vitelliform maculopathy [3], [22]. Some tissues of interest are only one or two voxels thick with even the most advanced clinically available OCT imaging technology. We have previously shown the accuracy of graph search segmentation compared to human experts [9], [23]. To be able to fairly compare two segmentation algorithms, i.e. the conventional approach and the non-Euclidean graph approach, we consider the segmentation of standard volumes at full resolution as the reference standard in this study. By down-sampling the full resolution volumes, we create “input volume data” which is segmented by the two approaches, and the resulting segmentation compared to the reference standard, allowing quantitative performance evaluation.

**Figure 8:**
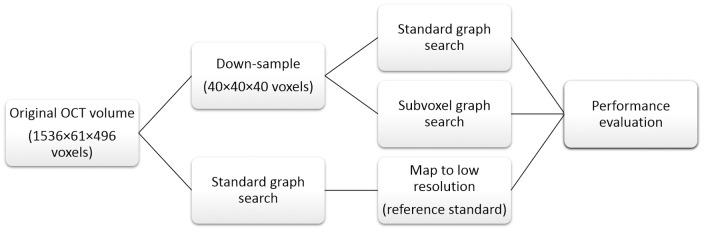
Experiment design for quantitative performance evaluation.

The process is illustrated in Fig. 8. Please note that in real applications, the high resolution volume and segmentation are unavailable. It is only used here to derive a reference standard for performance evaluation. All that can be obtained in real scenarios are the “input volume data”. Concurrently, the accuracy regarding to “voxel” or “sub-voxel” refers at the level of down-sampled voxels in this experiment. The absolute improvement of accuracy from voxel to subvoxel depends on the size of the voxel in physical dimension.

**Figure 9:**
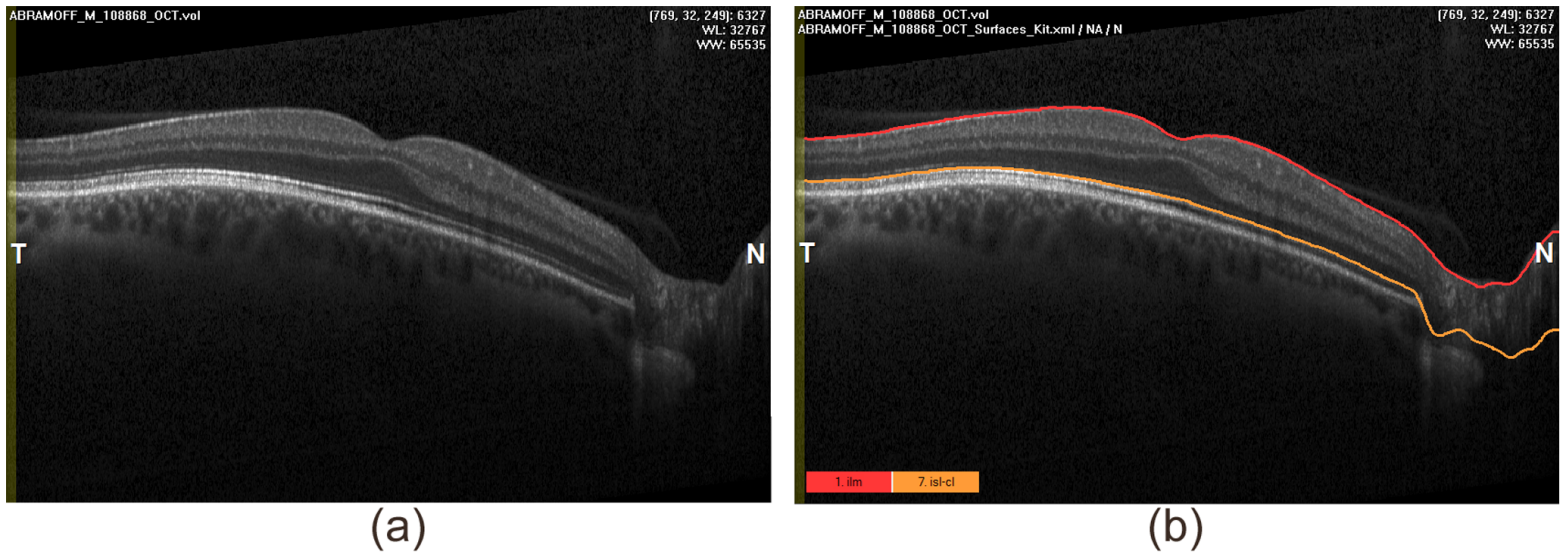
One B-scan of the high resolution OCT volume. (a) Original B-scan; (b) Two surfaces were identified.

In this study, we reused ten OCT volumes (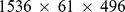 voxels, covering a region of 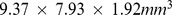 of the retina, Spectralis (Heidelberg, Germany)), from 10 eyes from 10 normal subjects as published previously by us [22] (available in deidentified form at http://webeye.ophth.uiowa.edu/abramoff/PLOSOne2014). The University of Iowa Institutional Review Board (IRB) approved the original study protocol and the reuse of data, the study was conducted according to the principles expressed in the Declaration of Helsink, and written informed consent was obtained from all subjects. As reference standard, two surfaces, both with dark-to-bright transitions, were identified using the Iowa Reference Algorithms, a publicly available implementation of the conventional graph search approach [3], [24]. One B-scan with the resulting segmentation is shown in Fig. 9.

**Figure 10:**
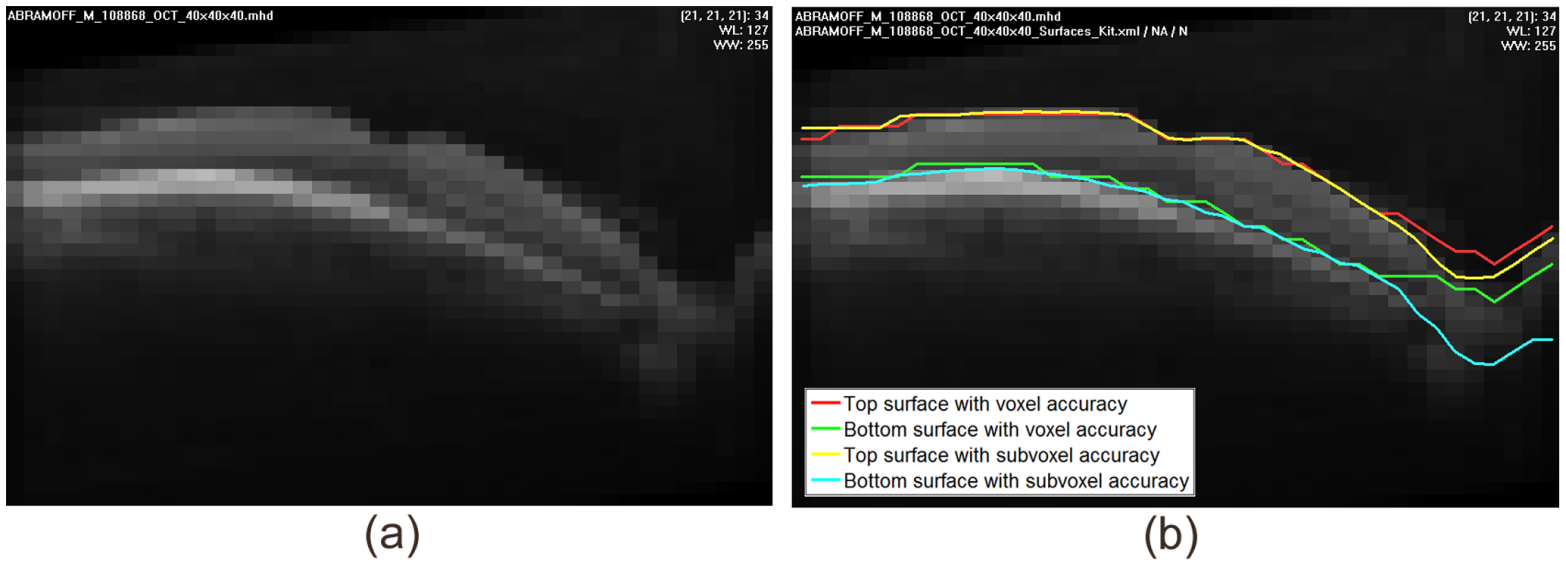
One B-scan of the down-sampled OCT volume. (a) One B-scan; (b) Two corresponding surfaces were identified by both conventional graph search and non-Euclidean graph search.

The original OCT volumes were down-sampled to 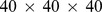 voxels, resulting in “input volume data”. The uneven down-sampling rate compensates for the highly anisotropic nature of typical OCT data and makes the partial volume effects clearly visible. The two surfaces were identified in these “input volume data” using the conventional graph search approach and our new non-Euclidean graph search approach (Fig. 10).

All other parameter settings were kept same in the two methods: the surface smoothness constraints between neighboring columns were set at one voxel, while the minimum and maximum surface separation constraint were at the usual 3 and 8 voxels respectively. The parameter 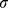 in the 3D Gaussian derivative kernel used to derive the deformation field and assign the cost was 0.3 voxel in all 3 dimensions. The choice of these parameters is primarily related to the resolution and aspect ratio of the data to be analyzed, and has always been used implicit in our previous work [3], [9], [10], [23], [25]. Though they were chosen for the Spectralis OCT volume data, we have not changed them from previous studies.

**Figure 11:**
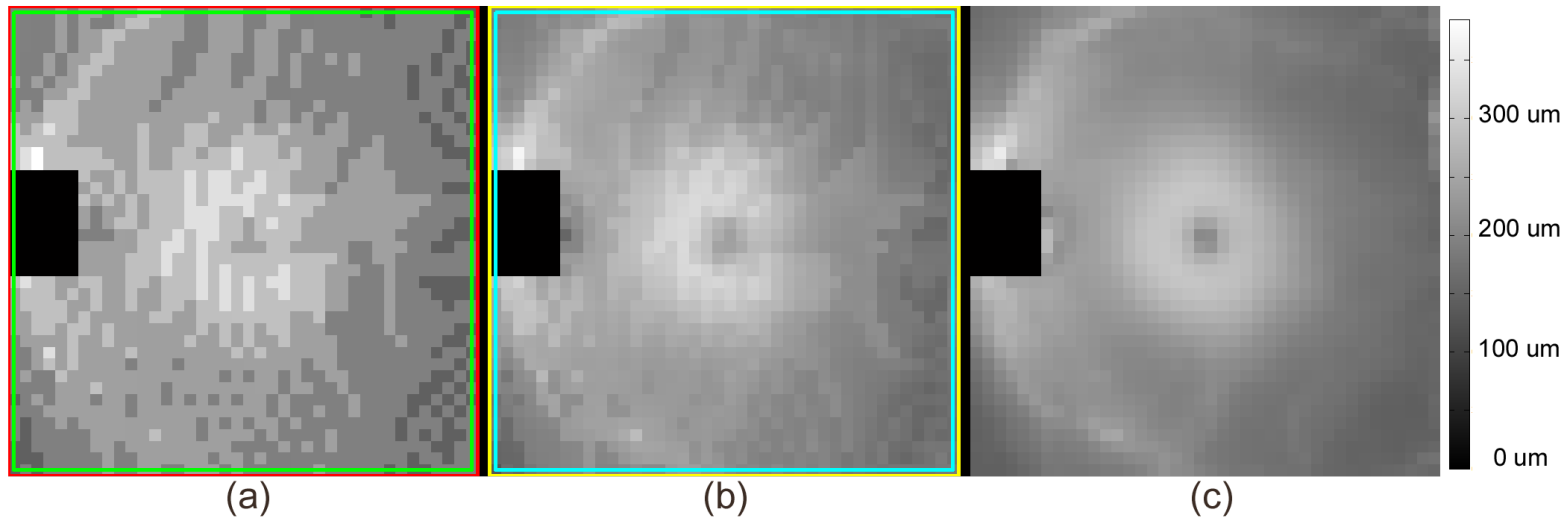
Example of tissue thickness map of the region bounded by two coupled terrain-like surfaces (the layer containing the outer segments of the retinal photoreceptors) segmented by (a) Conventional graph search of “input volume data” delineated by the red and green surfaces in Fig. 10 (b); (b) Non-Euclidean graph search of “input volume data” delineated by the yellow and cyan surfaces in Fig. 10 (b); (c) Mapping from segmentation results of the reference standard volume.

The upper surface 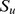 in Fig. 10 corresponds to the boundary of the inner limiting membrane (ILM) in Fig. 9 and the lower surface 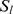 corresponds to the junction of the inner and outer photoreceptor segments, so that the layer itself corresponds to the assumed length of the outer segments of the photoreceptors [22]. Note that linear interpolation was applied to subvoxel surface segmentation result to get its location on an evenly distributed grid at each A-scan 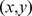. Thus it is possible to compute the thickness 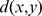 of the region bounded by those two surfaces 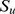 and 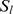 at each A-scan (Fig. 11) as:
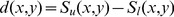
(8)


The layer thickness was most accurate in the map derived from the surfaces identified by our new method at subvoxel accuracy, which contains information that can not be approximated simply by any post-processing steps following conventional graph search. For example smoothing, which can also be observed from Fig. 1, resulted in less improvement, while requiring the assumption of monotonous thickness changes. The green surface located in Fig. 1 (b) is not a smoothed version of the red one in Fig. 1 (a). The former better reflects the imaged structure in presence of partial volume effects.

#### 0.3.3 Statistical Analysis

Both the mean signed 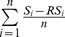 and unsigned 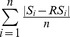 surface positioning errors (i.e. error or distance from ground truth) and layer thickness errors were calculated from the reference surface *RS* and then corrected for the fixed bias caused by the downsampling boundary shift. The difference between the mean errors for conventional graph search and non-Euclidean graph search was tested for significance using a paired t-test. Because the optic nerve head region is not a tissue layer and requires entirely different non surface segmentation approaches [25]–[27], a rectangular region of 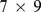 A-scans was excluded from analysis. The comparison is summarized in Table 1, where the accuracy of a smoothed version of conventional graph search is also listed as discussed above.

**Table 1:** Surface positioning error and thickness estimate error of region bounded by two coupled terrain-like surfaces (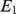: conventional graph search; 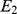: smoothed version of conventional graph search; 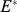: non-Euclidean graph search).

Errors in voxels	Top Surface 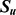 Error		Bottom Surface 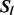 Error		Tissue Thickness 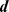 Error	
	Signed	Unsigned	Signed	Unsigned	Signed	Unsigned
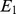 (conventional)	−0.04 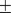 0.34	0.28 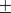 0.19	0.00 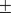 0.33	0.28 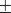 0.18	0.04 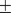 0.44	0.36 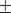 0.26
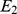 (smoothed conventional)	−0.07 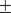 0.31	0.26 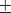 0.19	−0.04 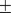 0.31	0.25 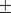 0.18	0.03 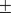 0.31	0.25 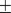 0.19
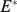 (non-Euclidean)	−0.02 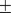 0.23	0.16 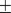 0.17	−0.03 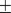 0.19	0.15 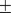 0.13	−0.01 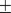 0.24	0.18 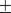 0.17
Paired t-test *p* ( 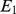 vs. 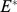 )	0.0180	0.0000	0.0000	0.0000	0.0000	0.0000
Paired t-test *p* ( 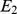 vs. 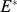 )	0.0912	0.0000	0.9311	0.0000	0.0010	0.0001

**Figure 12:**
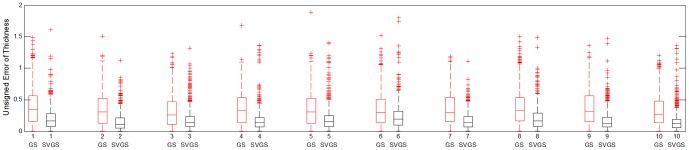
Box plot of unsigned error of tissue thickness among 10 subjects using conventional graph search (GS) and non-Euclidean graph search with subvoxel accuracy (SVGS).

For these 10 subjects, the unsigned errors were significantly smaller for non-Euclidean graph search than for both conventional graph search and its smoothed version with a p-value 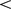 0.0001. The signed errors were not significantly different, because positive errors and negative errors cancel out when there are oscillations around the reference surface. The unsigned error of non-Euclidean graph search is almost always smaller than that of conventional graph search for the same subject at the same location. A box plot comparison of unsigned error of tissue thickness among all 10 subjects using conventional graph search and non-Euclidean graph search illustrates this in a different format in Fig. 12.

**Figure 13:**
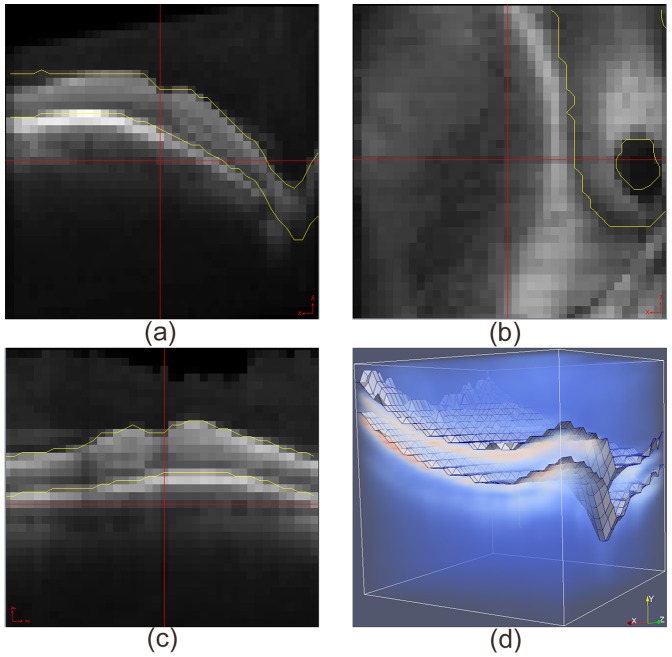
Segmentation of OCT volume with conventional graph search. (a) Cross section of one B-scan; (b) Top view; (c) Cross section perpendicular to B-scan; (d) 3D volume rendering.

**Figure 14:**
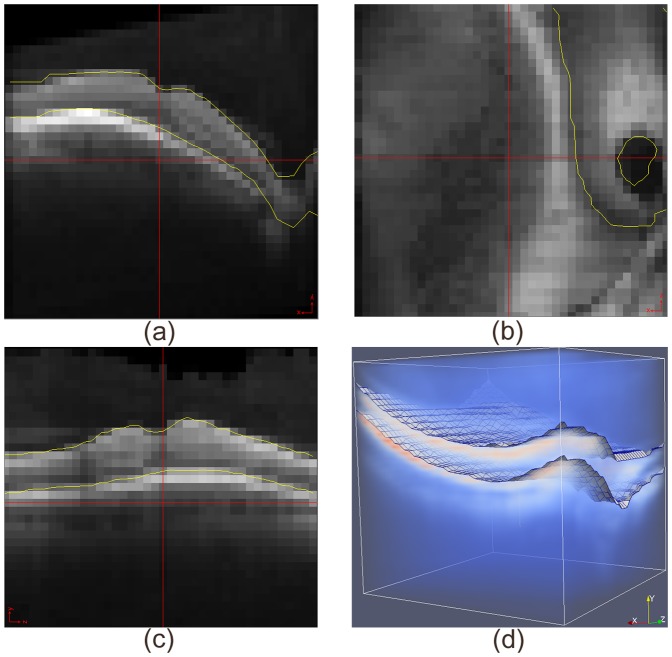
Segmentation of OCT volume with non-Euclidean graph search. (a) Cross section of one B-scan; (b) Top view; (c) Cross section perpendicular to B-scan; (d) 3D volume rendering.

Interestingly, the maximum difference between the two approaches is as large as 3.95 voxels even though the maximum displacement applied to each node of the non-Euclidean graph is not allowed to be more than half a voxel, as is shown in Fig. 10 (b). This is because all nodes encode potential boundary locations more precisely and the globally optimal solution is searched for in the new non-Euclidean graph space with each node taking on the minimum cost inside its corresponding voxel. More details are shown in Fig. 13 and Fig. 14.

**Figure 15:**
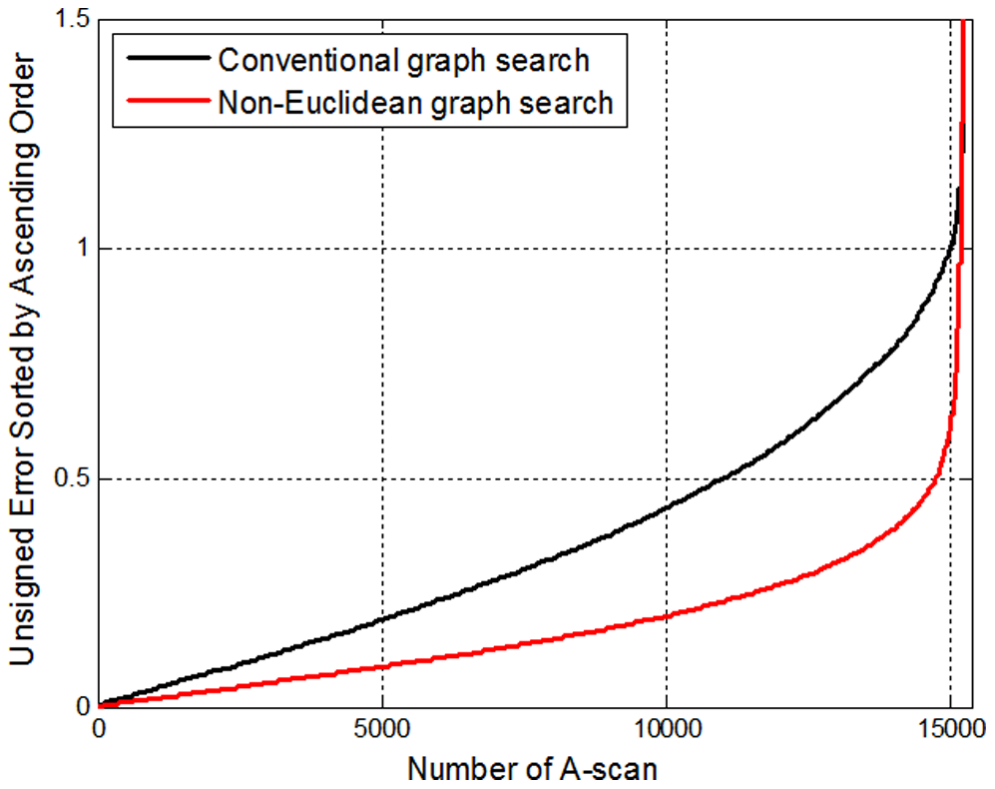
Comparison of unsigned error of tissue thickness produced by conventional graph search and non-Euclidean graph search among 15370 A-scans.

The unsigned thickness errors of both approaches over all 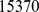 A-scans ((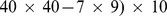)were sorted in ascending order and plotted in Fig. 15 for comparison. Non-Euclidean graph search reduces the percentage of A-scans with an error larger than 0.5 voxel from 27.25% to 3.14% compared to the conventional graph search method.

### Vascular Wall Segmentation Using 3-D MR Dataset

**Figure 16:**
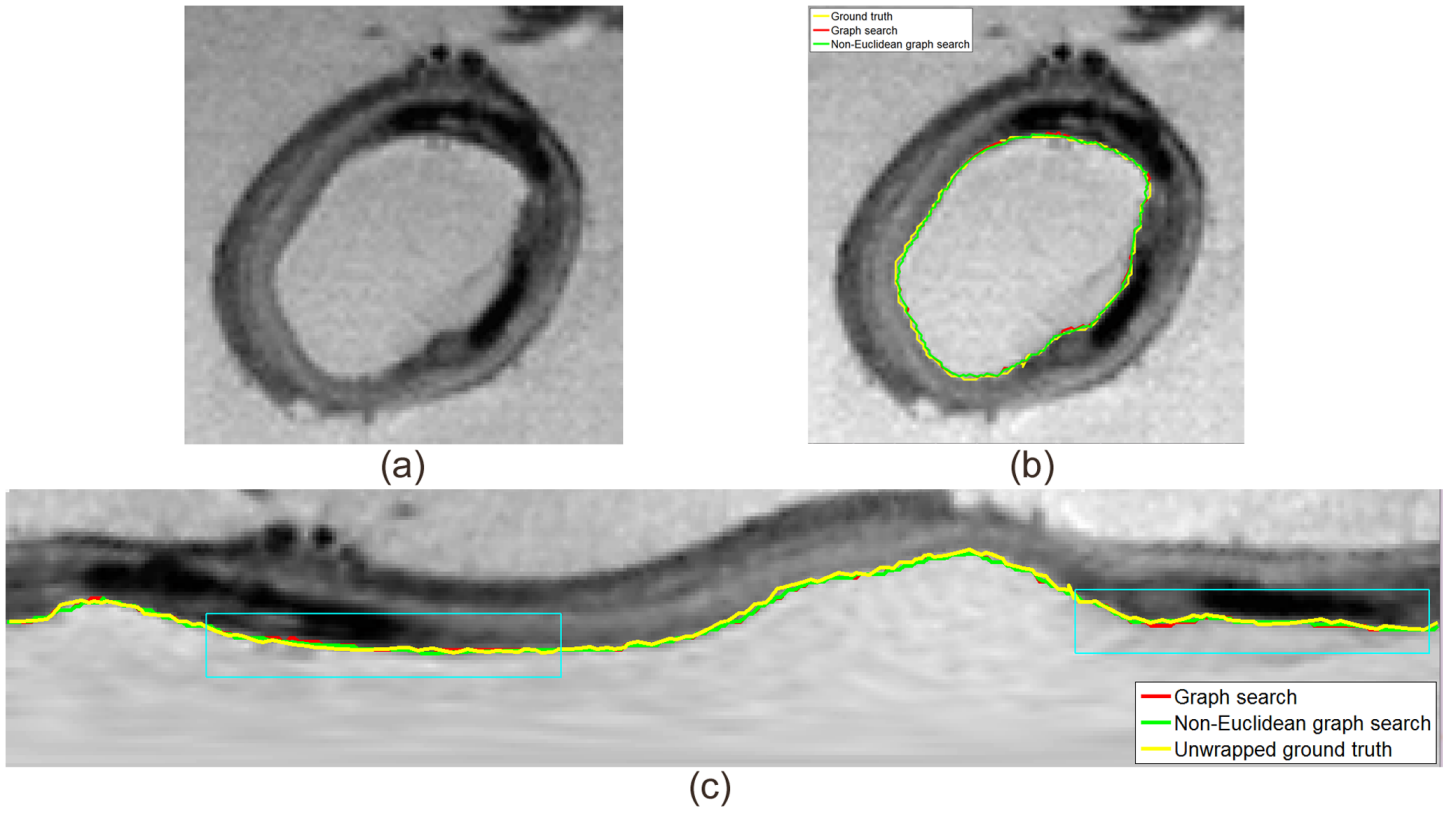
One slice of vascular wall segmentation by conventional graph search and non-Euclidean graph search. (a) Original slice; (b) Segmentation results; (c) Unwrapped slice. Result from the conventional graph search is shown in red, the non-Euclidean graph search in green and the ground truth in yellow.

**Figure 17:**
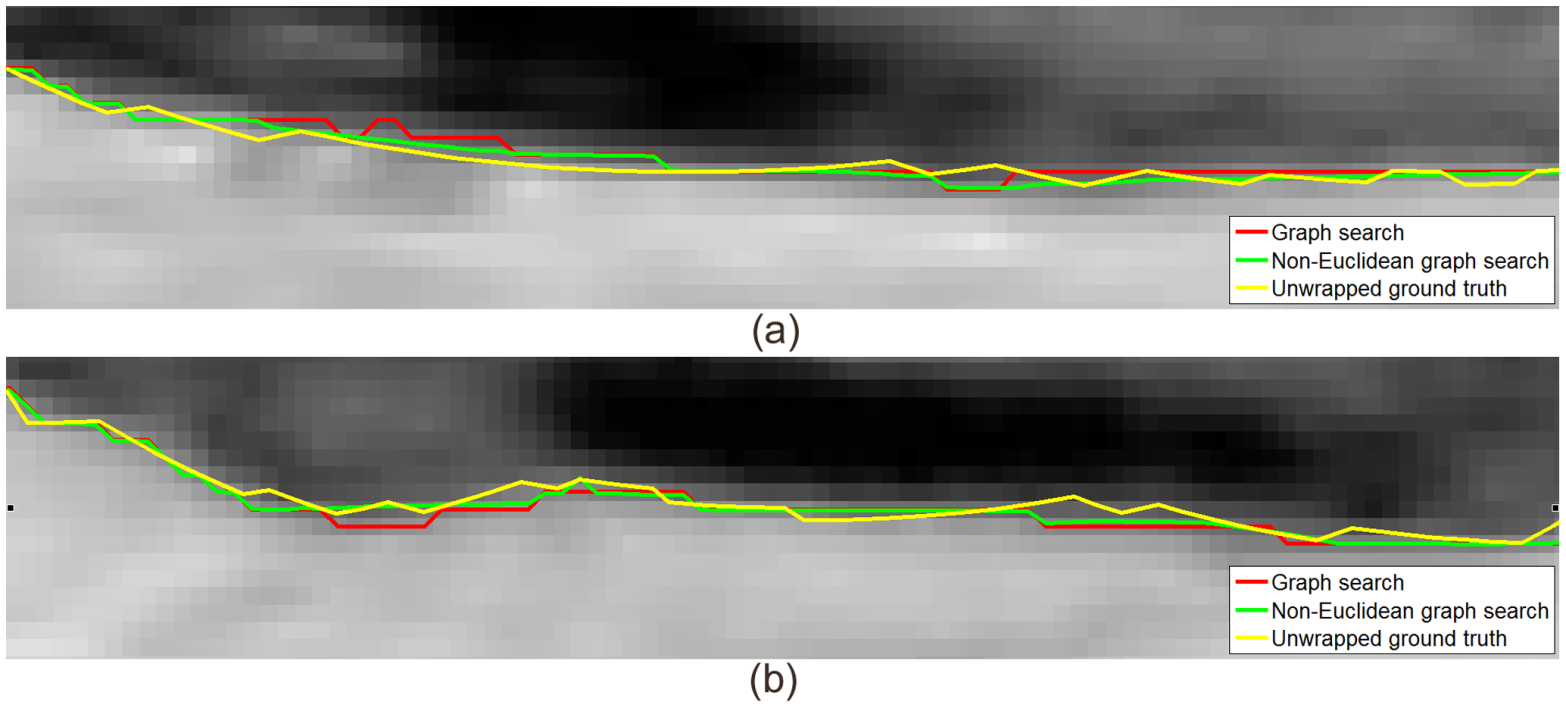
Zoomed in view of vascular wall segmentation results in the unwrapped slice. (a) the left region marked as a cyan rectangle in Fig. 16 (c); (b) the right region marked as a cyan rectangle in Fig. 16 (c).

The algorithm was also applied to identify the vascular wall – the lumen - intima surface, in 3-D MR image data already described in [5]. Two 3-D volumes with size of 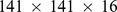 voxels were cylindrically unwrapped at the center of the volume to 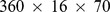 voxels. One terrain-like surface was identified using both conventional graph search and non-Euclidean graph search (Fig. 16). The surfaces were then mapped back to original volumes to highlight the cylindrical vascular wall (Fig. 16 (b)). The original volumes were manually annotated by an expert and superimposed in Fig. 16 (b) as reference. The manually traced boundaries were unwrapped and superimposed in Fig. 16 (c). More details were shown with two zoomed-in regions in Fig. 17.

The manual boundaries may not correspond exactly to the maximum intensity transitions at every location along the vascular wall due to its non-reproducible nature, thus preventing quantitative error measurements. Compared with conventional graph search, the surface identified by non-Euclidean graph search is closer to the manual delineation. Again, because the globally optimal solution is searched for in the new non-Euclidean graph space with each node taking on the minimum cost inside its corresponding voxel, the differences between the two approaches are larger than one voxel at a number of places even though the maximum displacement applied to each node is no more than half voxel.

## Discussion

We have generalized three-dimensional graph search to allow search in non-Euclidean graph space, and demonstrated that this results in increased segmentation accuracy, at a subvoxel level, for the surfaces of layered tissues. The graph is initially constructed in Euclidean space and then deformed using a deformation field so that the node density is increased near the expected surfaces, and decreased elsewhere. Because the total number of nodes and edges are unchanged, the memory requirement and running time are not affected, except for the time required to determine the deformation field from the volume data, which is negligible compared to the time spent for graph search. Our approach is agnostic with respect to the calculation of the deformation field, the only requirement is that it aligns node density with the inverse of the expected cost.

All advantages of graph search are thus retained including globally optimal surfaces in the time complexity of computing a single maximum flow [5] in polynomial time, the flexibility of combining various in-region and on-surface costs for simultaneous segmentation of multiple surfaces [9], and the capability of incorporating shape priors as in recent papers by Dufour and Song [8], [10].

We developed multi-scale graph search in order to limit memory requirements and run time required for multiple surface segmentation of large 3D volumes [25]. The non-Euclidean approach introduced here also supports multi-scale framework. At the finest scale subvoxel accuracy is achieved as under a regular framework.

The results on SD-OCT volumes of the retina and the vascular MR images of the vascular wall show that subvoxel precision is achieved and that segmentation accuracy compared to conventional search of the graph in Euclidean space is superior. In the present study, we demonstrated the improvements in accuracy using a non-Euclidean approach, using Optical Coherence Tomography and Magnetic Resonance Imaging based images. We would like to emphasize that our approach is not limited to these two modalities, but instead this approach is, in fact, very general and allows subvoxel accuracy in any multidimensional intensity based image where the intensities are the result of an imaging transform.

To our knowledge, this is the first attempt to construct the graph based on the underlying image content, using a generalized approach that allows non-equidistant representation of voxels on a single axis - hence the use of the term non-Euclidean for our approach. It is general in the sense that given any regular graph structure for segmentation, we can always achieve subvoxel accuracy by deforming that graph to a non-Euclidean one, regardless of imaging modalities or the underlying objects. This approach can be readily extended to higher-dimensional image segmentation, such as example 3D + time.

The advantages of increased, subvoxel accuracy while retaining memory and runtime requirements seem obvious, and allow either more accurate measurements in images obtained with the same image acquisition hardware, as well as, measurements with the same accuracy in images obtained at lower resolution, more cost-effective image acquisition hardware.
